# The relationship between coagulation abnormality and mortality in ICU patients: a prospective, observational study

**DOI:** 10.1038/srep09391

**Published:** 2015-03-23

**Authors:** Aihua Fei, Qiang Lin, Jiafu Liu, Feilong Wang, Hairong Wang, Shuming Pan

**Affiliations:** 1 Emergency Department, Xinhua Hospital, Shanghai Jiaotong University School of Medicine, Shanghai, China

## Abstract

We conducted a prospective, observational study to assess the prognostic value of hemostasis-related parameters in unselected ICU patients. We collected baseline characteristics from 497 consecutive unselected medical and trauma patients during their ICU stay. Each hemostasis-related parameter was analyzed alone or combined with APACHE II scores for any association with ICU mortality by calculating the under the curve (AUC) of the ROC curve, the net reclassification improvement (NRI) and integrated discrimination improvement (IDI) indices. Of all hemostasis-related indicators examined, the AUC for fibrin degradation products (FDPs) was less than that for APACHE II scores, but larger than that for disseminated intravascular coagulation (DIC) scores. The prediction power of FDPs is relatively low. Multiple regression analysis revealed that FDPs and APACHE II scores significantly predicted primary outcome. The combined use of FDPs level and APACHE II scores generated an NRI of 9.94% and an IDI of 3.54%. In conclusion, FDP is the best independent indicator of ICU mortality among all hemostasis-related indicators examined. The use of FDP level and APACHE II scores in parallel significantly improves the ability to predict ICU mortality, suggesting the application of these parameters could be used to improve patient care and management in the ICU.

Different types of scoring systems are used in intensive care units (ICUs) to predict morbidity and mortality of patients. Two of the most common scoring systems are the Acute Physiology and Chronic Health Evaluation II (APACHE II)^1^ and the Simplified Acute Physiology Score II^2^. These scoring systems are a highly useful tool for characterizing the diverse and heterogeneous nature of ICU patient groups, and in doing so, provides a prognostic prediction models for use in patient care and management. However, these models are generally calculated based on clinical scores, which often do not take into account a full spectrum of hemostasis-related parameters. The use of hemostasis-related parameters is important because coagulation abnormalities are commonly found in ICU patients, including thrombocytopenia, prolonged global coagulation, reduced levels of coagulation inhibitors, high levels of fibrin split products, and disseminated intravascular coagulation (DIC)^3^,^4^. In addition, critically ill patients are at an increased risk of developing thromboembolic complications, including deep vein thrombosis, pulmonary embolus, embolic stroke and myocardial ischemia.

The usefulness of hemostasis-related parameters in predicting clinical outcomes has been previously investigated in trauma patients. One retrospective study consisting of 314 trauma patients investigated the levels of hemostasis-related factors in patients immediately following arrival to the emerging department to up to 4 hour after arrival and found that JAAM DIC scores, levels of fibrinogen, fibrin degradation products (FDPs) and lactate are all independent predictors of mortality^5^. In this study, low levels of fibrinogen and high levels of FDP, but not D-dimers, predicted massive bleeding following trauma. Another study found that DIC scores, increased prothrombin time, FDPs and D-dimers accurately predicted mortality in patients with moderate or severe traumatic brain injury, whereas platelet counts and activated partial thromboplastin time (a-PPTK) only predicted mortality in those with severe injury^6^. Consistently, two studies found that prothrombin time (PT), FDP or D-dimer levels correlated with mortality in patients with traumatic brain injury^7^,^8^. These studies collectively indicate that certain hemostasis-related factors provide a solid indicator of wellbeing in patients following trauma.

Although it is clear that hemostasis-related parameters are reliable in predicting mortality in trauma patients, their prognostic use in patient care and management for all ICU patients is unclear. We, therefore, undertook a prospective, observational study to assess the potential use of a number of hemostasis-related parameters in predicting mortality in ICU patients. In addition, we evaluated whether the use of hemostasis-related parameters, combined with APACHE II scores, would improve mortality prediction in the ICU.

## Methods

### Participants

Consecutive adult patients admitted to the ICU of Xin-Hua Hospital affiliated with the Shanghai Jiaotong University School of Medicine, were enrolled in our study between April 2011 and June 2012. Eligible patients were those in need of intensive care treatment, who were transferred from the emergency department or other departments of our hospital, including medical and trauma patients (but not surgical patients). The decision to transfer patients to the ICU was made by at least one critical care expert and one medical or trauma expert. The decision to discharge or transfer patients from the ICU to general wards was made by the same expert panel. We formulated the following *a priori* exclusion criteria for our study: <18 years of age, pregnancy, and patients who died or were discharged within 4 hours of ICU stay (owing to difficulty in collecting complete dataset from these patients). Mortality corresponds to mortality during the length of the ICU stay. The study was approved by Shanghai Jiaotong University Xin Hua Hospital Ethics Committee (XHEC2011-011) and was carried out in accordance with the Declaration of Helsinki. Owing to the observational nature of this study and the fact that the laboratory indices used here are routinely collected from all patients admitted to our ICU, the need for written informed consent was waived by the ethics review board.

### Laboratory methods

Blood samples were obtained from patients admitted to the ICU for use in the measurement of platelet count, prothrombin time (PT), activated partial thromboplastin time (APTT), D-dimers, fibrinogen, fibrin degradation products (FDPs), and DIC scores. Blood samples were taken following admission to EICU of the hospital. Therefore, FDP levels were measured from blood taken after patient arrival to the EICU, not after diagnosis. Blood samples for blood count were collected into commercially available EDTA tubes. Plasma samples were collected into sample tubes (Sarstedt Nuembrecht, Germany) containing 0.106 M tri-sodium citrate (9:1). Samples were centrifuged for routine testing, and analysis was performed within 1 h after sampling.

Complete blood count and platelet count were determined using the Beckman Coulter LH-750 Hematology Analyzer (Beckman Coulter, Inc., Fullerton, California). Serum creatinine (SCr) and albumin were measured with the Hitachi 7600-120 Analyzer (Hitachi, Tokyo, Japan). We calculated the glomerular filtration rate (eGFR) using the abbreviated Modification of Diet in Renal Disease (MDRD) study equation: eGFR = 186 × (Scr)^−1.154^ × (Age)^−0.203^ × (0.742 if female).

All hemostasis-related parameters were determined using the ACL TOP 700 Analyzer (Instrumentation Laboratory Company, USA) and commercially available kits as well as reagents from the corresponding normal and pathologic control plasmas, and standard plasmas: PT-Fibrinogen HS PLUS for the PT, APTT-SP for the APTT. Prothrombin time and APTT are expressed as sec, Nanopia P-FDPs for FDPs,D-dimers HS 500 for D-dimers, and Scoring System for DIC. Coagulation variables at admission were scored according to different scoring systems, including the International Society of Thrombosis and Hemostasis (ISTH) DIC scores^9^, the Japanese Association for Acute Medicine (JAAM) scores, and revised JAAM DIC scores^10^.

HemosIL® D-dimer HS 500 is an automated, latex enhanced turbidimetric immunoassay for quantitative determination of D-dimer levels in human citrated plasma using the ACL TOP family systems^11^. The D-dimer HS 500 latex reagent is a suspension of polystyrene latex particles of uniform size coated with the F(ab′)2 fragment of a monoclonal antibody highly specific for the D-dimer domain included in fibrin soluble derivatives. The use of the F(ab′)2 fragment allows a more specific D-dimer detection, which avoids interference from rheumatoid factors. HemosIL® HS 500 works at a high wavelength (671 nm) where the effect of the optical interference is very low. The assay was tested for interference at two D-dimers concentrations, close to the cut off (600 ng/ml) and at a higher level (1700 ng/ml). The assay did not show interference for hemoglobin up to 500 mg/dl, bilirubin up to 18 mg/dl, and triglycerides up to 1327 mg/dl. Coated latex particles agglutinate when plasma containing D-dimers was mixed with the latex reagent and the reaction buffer. The degree of agglutination (i.e. the rate of light transmittance change) is directly proportional to the concentration of D-dimers and is determined by measuring the decrease in transmitted light caused by the aggregates. The results are reported in ng/ml of FEU. The linearity of the assay is 215–128,000 ng/ml with auto rerun.

The calibration curve was performed prior to the study and at frequencies recommended by the manufacturer using the kit calibrator (D-dimers partially purified from human fibrin digested with human plasmin). We analyzed two lyophilized controls (Low and High D-dimers, HS 500 Controls, Instrumentation Laboratory) in duplicate prior to analyzing plasma samples obtained from patients. If the controls fell out of specifications, the assay was re-calibrated and the controls re-tested until the results were within the specification. The clinical cut-off for VTE was 500 ng/ml.

FDP levels in plasma was determined quantitatively using a Latex immunoturbidimetry assay with commercial kit (Sekisui Chemical Co., Ltd., Osaka, Japan) as described previously^12^. The linearity of the assay is 0.25–12 g/L. The coefficient of variation from day to day ranges from 5% to 10% for pathologic plasmas.

### Statistical analysis

Continuous variables are presented as mean values ± SD or medians and ranges, and categorical variables are expressed as percentages. Variables were compared by an unpaired Student's t-test for continuous variables of normal distribution or the Mann-Whitney U test for continuous variables of non-normal distribution. A χ^2^ test or a Fisher's exact test was used for categorical variables. Because D-dimers and FDP values were of non-normal distribution, we used logarithmic transformation to analyze their linear relationship. Baseline characteristics between survivors and nonsurvivors were compared with an unpaired Student's t-test or the Mann-Whitney U test for continuous variables and a χ^2^ test or Fisher's exact test for categorical variables. Receivers operating characteristic (ROC) curves were used to examine the performance of variables in predicting ICU mortality. The area under the curve (AUC, also known as C-index) was calculated from the ROC curve. A statistically derived value based on the Youden's index that maximized the sum of the sensitivity and specificity was used to define the optimal cutoff value^13^. Univariate logistic regression analyses were performed to examine the association between mortality and individual predictors. We also conducted forward stepwise multivariate logistic regression analyses to determine the independent predictors of ICU mortality. Criteria of P < 0.05 for entry and P ≥ 0.10 for removal were imposed in this procedure. Cox & Snell R2 and Nagelkerke R2 correlation coefficients were calculated to assess goodness of fit in the models. ORs for continuous variables were described with standard ORs, which were associated with a 1-SD change in the variable. The increased discriminative predictive value of the FDPs level and the APACHE II scores combination was examined by calculation of net reclassification improvement (NRI) and integrated discrimination improvement (IDI) indices as described by Pencina, et al^14^. NRI is the net increase vs. the net decrease in risk categories among case patients minus that of the control participants. It requires that there exist *a priori* meaningful risk categories (we used less than 5%, 5–10%, 10–20% and more than 20% for the risk of ICU death). IDI is the difference in Yates slopes between models, where the Yates slope is the mean difference in predicted probabilities between case patients and control participants^15^. A two-sided P value less than 0.05 was considered statistically significant. All analyses were performed using SPSS version 13.0 software (SPSS, Inc, Chicago, IL, USA).

## Results

### Baseline characteristics of the study cohort

We enrolled 497 consecutive patients in this study, of those 64% were male. The mean age of the patients was 67 ± 17 years. The primary reasons for EICU admission were cardiovascular disease and pulmonary disease. The median level of D-dimers, FDPs, DIC by ISTH, DIC by JAAM, and DIC by revised JAAM on admission was 0.61 (range: 0.010 to 20.00) mg/L, 4.40 (0 to 429) mg/L, 1(0 to 5), 2 (0 to 9) and 1 (0 to 8), respectively. The mean APACHE-II scores were 15.57 ± 8.15 points. A total 36% of the patients had accompanying infections. Our study includes patients who were treated with anticoagulant therapy. Included in our analysis were those with cardiovascular disease (n = 137; 27.6%) who were treated with anticoagulant therapy. We did not observe a noticeable difference between patient groups treated or not treated with anticoagulant therapies.

Non-survivors were older, however, this was not statistically significant. These patients were associated with more severe conditions, had higher APACHE II scores, suffered infection more frequently, and had hemostatic abnormalities, including prolonged PT and APTT, higher D-dimers and FDPs. Non-survivors had higher white blood cell counts and heart rates, and lower eGFRs and blood pressure on admission. All baseline clinical and laboratory characteristics of the patients are shown in Table 1.

### The predicting value of indicators for ICU mortality

We performed univariate logistic regression analyses to examine the association between ICU mortality and different clinical indicators by calculating the standardized coefficient (b) and OR for each variable (Table 2). Univariate logistic regression analyses demonstrated that older patients with higher APACHE II scores, CRP, D-dimers, FDPs, DIC scores, and prolonged PT and APTT, were significantly associated with mortality (Table 3). In addition, lower eGFR and lower platelet counts were also associated with significantly greater mortality (Table 3). The absolute value of standardized b (0.93) of D-dimers was less than that of APACHE II (1.30), however, it was the highest among all coagulation indicators. Revised JAAM DIC scores had greater absolute value of standardized b (0.71) than that of ISTH DIC scores (0.66) or original JAAM DIC scores (0.67). To examine the performance of indicators as predictors of ICU mortality, ROC curves were constructed to allow the calculation of AUC for each indicator. The AUC, optimal cutoff value, sensitivity and specificity of each indicator are given in Table 3. The AUC for APACHE II was 0.84 ± 0.05, which was the highest among all indicators. For blood coagulation indicators, D-dimers had the greatest power for predicting ICU mortality, as shown by the largest AUC (0.76 ± 0.06), followed by FDPs (0.74 ± 0.06) and PT (0.70 ± 0.07). New DIC scores (AUC = 0.73 ± 0.06) were a better predictor of ICU mortality than ISTH DIC scores (0.68 ± 0.08), original JAAM DIC scores (0.70 ± 0.07) and revised JAAM DIC scores (0.71 ± 0.07).

### Predictive value of FDPs and APACHE II in a multivariate model

We conducted forward stepwise multivariate logistic regression analyses to determine independent predictors of ICU mortality. Among coagulation indicators, DIC scores, APACHE II scores and FDPs were the only independent predictors accepted by the prediction models (P = 0.003).

The optimal cutoff value of APACHE II scores for predicting death was ≥16, which provided a sensitivity of 81.5% and a specificity of 71.5%. The optimal cutoff value of FDP was ≥ 3.3(mg/L), which provided a sensitivity of 87.7% and a specificity of 47.7%.

**Figure 1:**
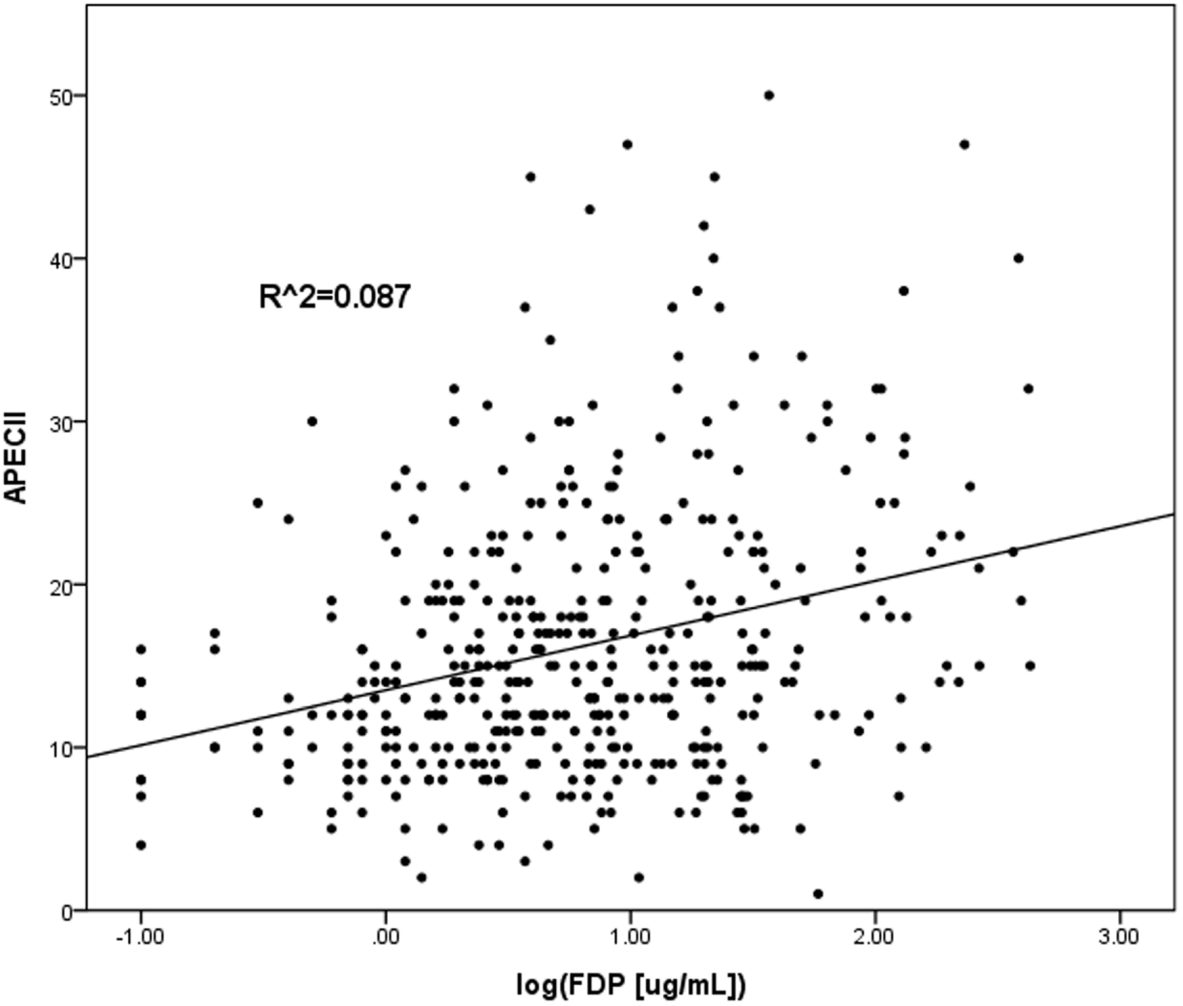
The correlation between FDP and APACHE II Scores. A log scale of FDP and APACHE II scores R2 was generated and is 0.087. FDP: plasma fibrin and fibrinogen degradation products; APACHE II score, Acute Physiology and Chronic Health Evaluation II score.

**Figure 2:**
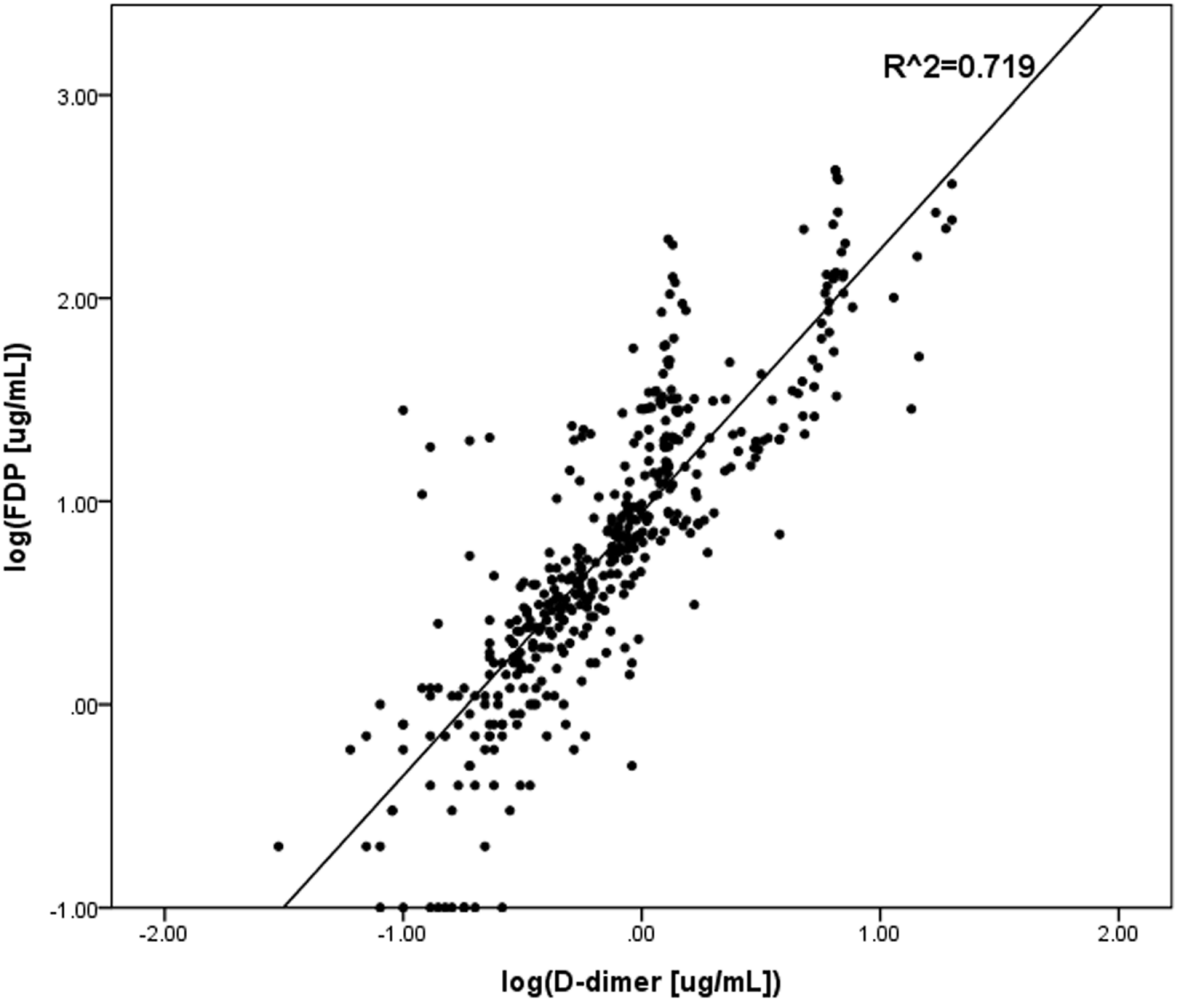
The correlation between FDP and D-dimers. A log scale of FDP and D-dimers values R2 was generated and is 0.719, indicating a strong correlation. FDP: plasma fibrin and fibrinogen degradation products.

A simple linear regression, using log transformed FDPs and log transformed D-dimer values from a total of 497 patients, yielded a correlation coefficient (R^2^) of 0.79 (Fig. 1), whereas R^2^ of log transformed FDPs and APACHE II scores was 0.09 (Fig. 2), indicating additional predictive value of FDPs from APACHE II scores.

### Combined use of FDP and APACHE II scores for predicting ICU mortality

**Figure 3:**
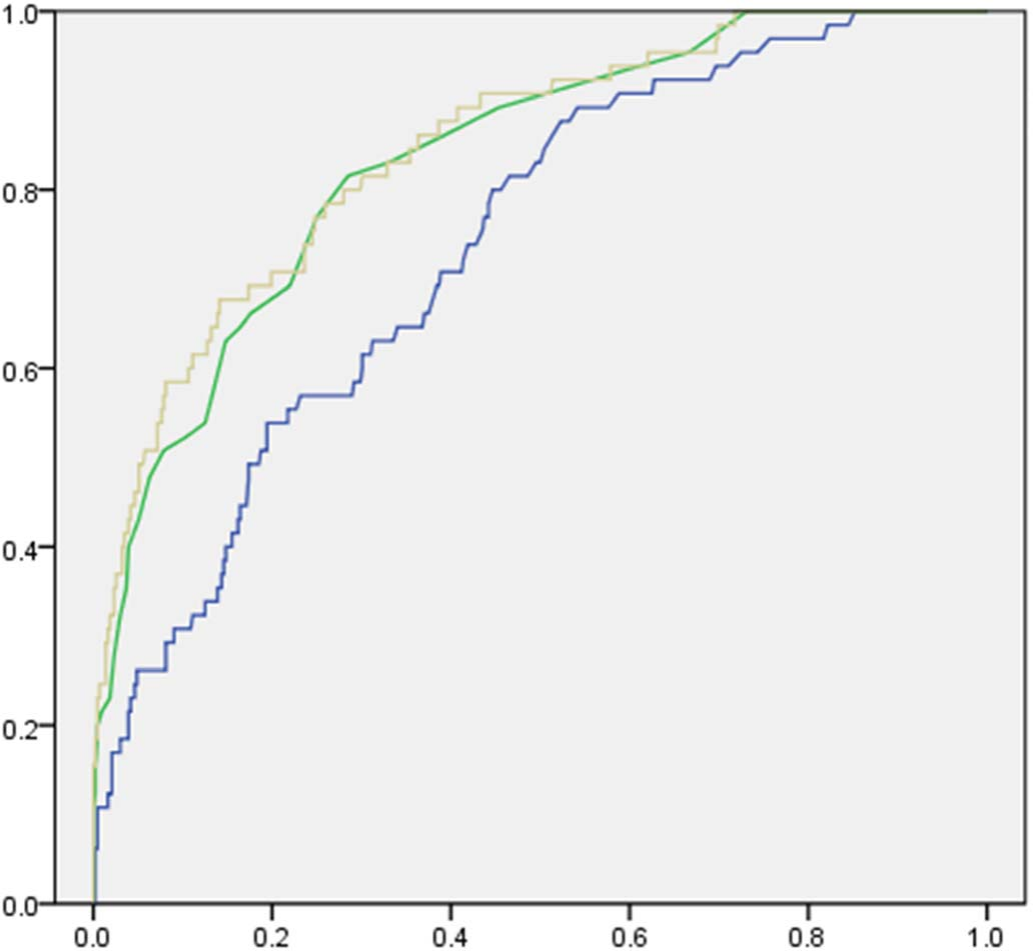
ROC curves generated by the use of FDP and APACHE II scores in the same model. Green: APACHE II scores. Brown: FDP with APACHE II scores. Blue: Prothrombin time.

To further investigate whether FDP enhances the predictive power of APACHE II scores for ICU mortality, we used FDP and APACHE II scores in the same model to construct new ROC curves (Fig. 3). As compared with APACHE II scores (AUC 0.84 ± 0.02), the combined use of FDP (AUC 0.85 ± 0.02) with APACHE II scores did not significantly increase AUC for predicting ICU mortality (P = 0.74). However, forward stepwise logistic regression showed that of the combined use of FDP and APACHE II scores slightly increased the ability of the model to predict ICU mortality. The Cox & Snell R Square and Nagelkerke R Square were slightly increased in the model (Table 4). When using NRI and IDI indices for statistical analysis, methods that are more sensitive than the above statistical analysis, we found that the combined use of FDP and APACHE II scores significantly improved the predictive capacity of ICU mortality compared to the use of APACHE II scores alone (Table 5). The combined use of FDP and APACHE II scores generated an IDI of 3.5% (P = 0.03) and NRI of 9.9% (P = 0.04). In the non-survivor group, 9.23% (n = 6) of the patients were reclassified by FDP to a higher risk category and 4.6% (n = 3) to a lower risk category. In the survivor group, 3.5% (n = 15) of the patients were reclassified by FDP to a higher risk category and 8.1% (n = 35) to a lower risk category.

## Discussion

Hemostasis-related parameters are reliable indicators for use in predicting mortality in trauma patients, however, their prognostic value in patient care and management for all ICU patients is unclear. Here we investigated in 497 unselected ICU patients whether hemostasis-related parameters can be used to accurately predict ICU mortality. We found that of all hemostasis-related parameters investigated, FDPs was able to independently predict ICU mortality even after adjustment for the APACHE II scores and multiple confounders, including eGFR, age, and other coagulation parameters. It is important to note that the association between eGFR and mortality could be a consequence of the greater age of the non-survivors in our study cohort. The clinical implication of our study is that combined use of the FDPs level and APACHE II score allows a better prediction of mortality, which could be used to optimize patient care and management in the ICU.

FDPs are regarded as one of the most useful parameter to indicate lysis of fibrin deposits^16^, especially for the diagnosis of DIC and potential acute aortic dissection^17^. In a study involving 314 severe trauma patients, Sawamura et al. found that JAAM DIC scores, levels of fibrinogen, FDPs and lactate were independent predictors of death^5^. To our knowledge, the predictive capacity FDPs in mortality has not been investigated in ICU patients. Here, we report, for the first time, that FDPs could be used for predicting ICU mortality. However, the prediction power of FDPs is relatively low (AUC of 0.74 ± 0.06, P < 0.001), with a sensitivity of 87.7% and a specificity of 47.7%. Furthermore, the predictive ability of FDP alone was lower than that of the APACHE II scores. However, we identified that the combined used of FDPs and APACHE II scores significantly improves the accuracy in predicting ICU mortality. Indeed, ROC curves in Figure 2 show that combining FDPs and APACHE II scores increases the ability to discriminate mortality risk. The newly developed statistical analysis methods generated an IDI of 3.5% (P = 0.04) and a NRI of 9.9% (P = 0.04), which confirmed the superior performance of FDPs and the APACHE II scores in predicting ICU mortality.

D-dimers, a component of FDPs, have been reported to be associated with mortality of ICU patients. Kollef et al. measured D-dimers using a semi-quantitative, rapid enzyme-linked immunosorbent assay (ELISA) in 321 MICU patients and reported that in-hospital mortality was significantly higher in D-dimers positive patients (p = 0.004)^18^. The levels of D-dimers also correlated with the development of MSOF and sepsis. Shorr et al. employed a latex agglutination assay to measure D-dimers and found that critically ill patients with a positive D-dimers test have an increased risk of in-hospital death and venous thromboembolism (VTE)^19^. Consistently, our study showed that D-dimers have the greatest prediction power for ICU mortality (AUC 0.76 ± 0.06; P < 0.001; sensitivity 78.5%; specificity 63.2%), however, it is not an independent factor for ICU mortality after adjustment. Although there is a strong correlation between FDP and D-dimers in our study, it is not unusual to observe that only FDP was associated with mortality. FDPs are fibrin degradation products produced from clots or from circulating fibrinogen, whereas D-dimers are cross-liked fibrin degradation products more specifically produced as a result of breakdown of clots. It is possible that the association between FDPs levels and mortality indicates that the degree of clot remodeling (as indicated by levels of D-dimers) is not a major contributor to mortality.

Several studies have found significant correlations between DIC scores and scoring systems in ICUs, such as Sequential Organ Failure Assessment (SOFA) and APACHE II. Satoshi Gando et al. identified that revised JAAM criteria are a significant independent predictor of death^10^. Angstwurm and co-workers showed that increasing coagulation score was associated with increasing mortality and decreasing survival rates^20^. In addition, this study found that combined use of APACHE II scores and the scoring system for DIC predicts mortality in critically ill patients better than the APACHE II scores alone. However, our results did not support these observations. DIC scores by JAAM, revised JAAM, ISTH, and new DIC scores were predictors of death following univariate logistic regression analysis, however, none of these were independent after revision. The differences in observation between our study and that of Angstwurm and colleagues may be due to several factors. Our results were derived from a prospective study, while that of Angstwurm et al. was from a retrospective study. In addition, differences in gerographic regions (e.g. China in our study and Germany in the study by Angstwurm et al.) and differences in the methods of data collection and analysis may influence the final outcome.

In our study, we did not investigate the causes of elevated FDPs, including Thrombotic Microangiopathy (TMA), VTE, and specific types of cancer, which may often confound the relationship between FDPs and adverse outcomes. To overcome this we adjusted several other potential confounders, including DIC scores and APACHE II scores. We were also aware that routine coagulation tests cannot evaluate certain coagulation abnormalities. Findings from this single-center study would require confirmation from prospective studies from other centers. In addition, it would be useful to observe the dynamics for the production of FDPs, since it remains unclear whether FDPs levels are elevated in a time-dependent manner when a patient's condition progressively deteriorates. Quality of life and other endpoints after discharge can't be evaluated because our patients were not followed after leaving the ICU. Unfortunately, we also do not have data on previous hospital admissions in these patients, therefore, we are unable to quantify the proportion of risk attributable to FDPs and APACHE II scores.

## Conclusion

Coagulation abnormality is common in ICU patients. Our results suggest that of all routine coagulation tests, FDP concentration is a prognostic biomarker associated with poor survival in our ICU population, which provides a useful tool for use in parallel with APACHE II scores for predicting ICU mortality.

## Author Contributions

A.F., Q.L. and S.P. designed of the experiments; S.P., A.F., Q.L., J.L. and F.W. performed the experiments; S.P., A.F., Q.L., J.L., F.W. and H.W. collected the date; S.P., A.F., Q.L., J.L. and H.W. wrote the main manuscript text and all authors reviewed the manuscript.

## Figures and Tables

**Table 1:** Baseline clinical and laboratory characteristics of the study subjects

Characteristics	All(497)	Survivors(432)	Non-survivors(65)	P values
Gender (%)				0.795
Male	319(64.2)	276(63.8)	43(66.2)	
Female	178(35.8)	156(36.2)	22(33.8)	
Principal diagnosis leading to ICU (%)				0.335
Cardiovascular disease	137(27.6)	123(28.5)	14(21.5)	
Neurologic disease	79 (14.8)	64(14.8)	15(23.1)	
Pulmonary disease	93(18.7)	82(19.0)	11(16.9)	
Digestive disease	49(9.9)	43(10.0)	6(9.2)	
Poisoning	20(4.0)	19(4.4)	1(1.5)	
Trauma	21(4.2)	16(3.7)	5(7.7)	
Other	98(19.7)	85(19.7)	13(20)	
Accompanying infection (%)				0.349
Yes	188(62.2)	160(37.0)	28(43.1)	
No	309(37.8)	272(63.0)	37(56.9)	
Age (years)	67.28 ± 17.24	66.64 ± 17.58	71.55 ± 14.23	0.054
Platelet	165.50 ± 72.20	168.86 ± 71.20	143.15 ± 75.32	0.533
eGFR (mL/minute/1.73 m^2^)	82.56 ± 50.16	84.13 ± 42.77	72.08 ± 83.95	<0.001
PT (second)	13.23 ± 4.59	12.82 ± 3.95	15.89 ± 7.05	<0.001
PT/INR	1.16 ± 0.30	1.12 ± 0.26	1.34 ± 0.46	<0.001
APTT (second)	31.71 ± 9.15	31.23 ± 8.03	34.90 ± 14.24	<0.001
Fibrinogen (g/L)	3.74 ± 1.50	3.79 ± 1.44	3.43 ± 1.80	0.005
Thrombin time (second)	15.42 ± 4.68	15.23 ± 4.57	16.71 ± 5.22	0.090
CRP (mg/L)	25.0(6.0 to 160.0)	21.0(6.0 to 160.0)	57.0(8.0 to 160.0)	<0.001
FDP (mg/L)	4.40(0 to 429)	3.75(0 to 429)	19.9(0.20 to 421.6)	<0.001
D-dimers (mg/L)	0.61(0.01 to 20)	0.53(0.10 to 18.85 )	1.27(0.19 to 20)	<0.001
APACHE II score	14 (1 to 50)	13(1 to 40 )	25 (10 to 50)	<0.001
ISTH	1 (0 to 5)	0 (0 to 5)	2 (0 to 5)	<0.001
JAAM	2 (0 to 9)	2 (0 to 9)	3 (0 to 8)	<0.001
rJAAM	1 (0 to 8)	1 (0 to 8)	1 (3 to 8)	<0.001

APACHE II score, Acute Physiology and Chronic Health Evaluation II score; APTT Apartial thromboplastin time; CRP, C-reactive protein; eGFR, estimated glomerular filtration rate; FDP, Fibrinogen degradation product; INR International Normalized Ratio; PT, Prothrombin time.

**Table 2:** Hemostasis-related independent predictors of ICU mortality

Variable	OR(95%CI)	Standardized Estimate	P values
PT	1.093(1.041, 1.147)	0.223	<0.001
Unit = 2	1.195		
FDP	1.011(1.006, 1.015)	0.322	<0.001
Unit = 10	1.114		
Age	1.027(1.006, 1.048)	0.249	0.012
Unit = 5	1.142		

The Risk ratio was estimated by using a stepwise multivariate logistic regression model, with PT, APTT, fibrinogen, age, thrombin time, D-Dimers, FDP, Principal diagnosis disease as covariates.

**Table 3:** ROC of ICU mortality for Hemostasis-related parameters and scoring systems

Variable	AUC(95%CI)	Cutoff value	Sensitivity	Specificity	Youden's index	P values*
CRP	0.645(0.580, 0.710)	39.50	0.646	0.356	0.002	
Fibrinogen	0.431(0.346, 0.515)	4.68	0.292	0.759	0.051	
APTT	0.563(0.481, 0.645)	36.35	0.277	0.860	0.137	
PT/INR	0.704(0.633, 0.774)	1.07	0.846	0.487	0.333	
PT	**0.704(0.633, 0.774)**	12.00	0.831	0.508	0.339	0.002
FDP	**0.736(0.676, 0.797)**	3.35	0.877	0.477	0.354	0.004
D-dimers	**0.755(0.697, 0.813)**	0.82	0.785	0.632	0.417	0.016
APACHE II score	**0.835(0.784, 0.886)**	16.00	0.815	0.715	0.53	
JAAM	0.702(0.632, 0.771)	2.50	0.6	0.704	0.304	
rJAAM	0.710(0.639, 0.782)	2.50	0.554	0.773	0.327	
ISTH	0.725(0.653, 0.797)	1.00	0.723	0.622	0.345	

APACHE II score, Acute Physiology and Chronic Health Evaluation II score; APTT Apartial thromboplastin time; CRP, C-reactive protein; FDP, Fibrinogen degradation product; INR International Normalized Ratio; ISTH, International Society of Thrombosis and Hemostasis (ISTH) DIC scores; JAAM the Japanese Association for Acute Medicine DIC score; PT, Prothrombin time; rJAAM revised JAAM DIC scores.

*AUC Compared with APACHE II scores.

**Table 4:** Independent predictors of ICU mortality by multivariate logistic regression in all patients (appending models summary)

		OR	OR-st	*P*	−2 Log likelihood	Cox & Snell R Square	Nagelkerke R Square
**Model I**	APACHE II score	1.178	3.596	0.000	280.141	.177	.330
**Model II**	LogFDP	1.008	3.175	0.001	268.355	.197	.367
	APACHE II score	1.169	1.702	0.000			

APACHE II score, Acute Physiology and Chronic Health Evaluation II score; FDP, Fibrinogen degradation product; OR, odds ratio; OR-st, standardized Odds ratios (OR per 1 SD).

**Table 5:** ROC of ICU mortality for combined scoring systems

Variable	AUC(95%CI)	Cutoff value	Sensitivity	Specificity	Youden's index	P values*
APACHE II score	0.835(0.784, 0.886)	16.5	0.815	0.715	0.53	
FDP + APACHE II score **	0.849(0.798, 0.900)	21.5	0.815	0.762	0.577	0.087
PT + APACHE II score**	0.854(0.809, 0.900)	21.5	0.785	0.762	0.547	0.031
FDP + PT + APACHE II score**	0.865(0.819, 0.910	26.5	0.785	0.817	0.602	0.006

*AUC Compared with APACHE _II scores;

**The score weight of FDP and PT added to APACHE II scores was 5 when they were above the cutoff value in Table 3.
